# Prevention of Hospital-Acquired Venous Thromboembolism in Children: A Review of Published Guidelines

**DOI:** 10.3389/fped.2017.00009

**Published:** 2017-01-26

**Authors:** E. Vincent S. Faustino, Leslie J. Raffini

**Affiliations:** ^1^Department of Pediatrics, Yale School of Medicine, New Haven, CT, USA; ^2^Division of Hematology, The Children’s Hospital of Philadelphia, Philadelphia, PA, USA

**Keywords:** child, deep venous thrombosis, guideline, heparin, pulmonary embolism, prophylaxis, randomized controlled trial

## Abstract

Venous thromboembolism, which includes deep venous thrombosis and pulmonary embolism, is a potentially preventable condition in children. In adults, pharmacologic prophylaxis has been shown to significantly reduce the incidence of venous thromboembolism in distinct patient cohorts. However, pediatric randomized controlled trials have failed to demonstrate the efficacy of pharmacologic prophylaxis against thrombosis associated with central venous catheters, the most important risk factor for venous thromboembolism in children. Despite the lack of supporting evidence, hospital-based initiatives are being undertaken to try to prevent venous thromboembolism in children. In this study, we sought to review the published guidelines on the prevention of venous thromboembolism in hospitalized children. We identified five guidelines, all of which were mainly targeted at adolescents and used various risk-stratification approaches. In low-risk children, ambulation was the recommended prevention strategy, while mechanical prophylaxis was recommended for children at moderate risk and pharmacologic and mechanical prophylaxis were recommended for the high-risk group. The effectiveness of these strategies has not been proven. In order to determine whether venous thromboembolism can be prevented in children, innovative clinical trial designs are needed. In the absence of these trials, guidelines can be a source of valuable information to inform our practice.

## Background

In the past decade, the incidence of venous thromboembolism (VTE) in children has steadily increased. On average, the incidence increased by nearly 10% per year (1). VTE, which is composed mainly of deep venous thrombosis (DVT) and pulmonary embolism (PE), is associated with prolonged duration of mechanical ventilation and prolonged stay in the pediatric intensive care unit (PICU) and in the hospital (2). The excess hospital stay drives the increased cost associated with VTE in hospitalized children (3). In some cases, VTE may lead to death (4). The increasing incidence of VTE in children is thought to be the result of improved survival in critically ill children (1). It is also likely that a heightened awareness of VTE has contributed to increased diagnosis.

Hospital-acquired VTE is preventable in adults (5–7). Multiple randomized controlled trials (RCTs) in adults have demonstrated the superiority of pharmacologic prophylaxis, compared to placebo, in reducing the incidence of VTE in targeted high-risk populations. It is possible that VTE is also preventable in children. In fact, the Solutions for Patient Safety network, a national collaborative of children’s hospitals in the United States, has aimed to reduce harm from hospital-acquired VTE (8). However, in contrast to adults, data are lacking to support the use of pharmacologic prophylaxis in children. Pediatric RCTs of prophylaxis have focused on central venous catheter (CVC)-associated DVT (CADVT) because this is the most important cause of DVT in children. In a systematic review of RCTs of CADVT, Vidal et al. analyzed two RCTs on heparin-bonded CVC, three on infusions of unfractionated heparin at doses of ≤10 U/kg/h, one on reviparin [a low molecular weight heparin (LMWH)] at prophylactic dose, one on warfarin, one on antithrombin concentrate, and one on nitroglycerin (9). None of them were efficacious. Relative risks of CADVT with intervention ranged from 0.06 to 1.53, none of which were statistically significant. Despite the lack of supporting evidence, hospital-based initiatives are being undertaken to try to prevent VTE in children. In this study, we sought to review the different published guidelines on the prevention of VTE in hospitalized children.

## Methods

We searched MEDLINE (OvidSP) for studies from inception of the database until September 2016. With a medical research librarian, we developed, refined, and performed the search. We searched for VTE AND prevent* AND (child* OR pediatr*) AND (algorithm* OR protocol* OR guideline*). We also hand searched our personal lists and references of eligible studies. From the list of articles, we identified those that reported on a guideline for the prevention of DVT in children. Salient characteristics of each guideline including target population, risk factors considered, intervention, compliance, and frequency of DVT and bleeding were abstracted from the full text of the article (Tables 1 and 2).

**Table 1:** **Characteristics of published guidelines for thromboprophylaxis in children**.

Reference	Target population	Risk categories	Criteria	Interventions
Braga and Young (10)	Children in the pediatric intensive care unit (PICU)	Low risk	Immobility	Early mobilization and adequate hydration
		At risk	≥1 additional risk factor for VTE	Mechanical prophylaxis, pharmacologic prophylaxis to be considered for children with burns

Raffini et al. (11)	Children ≥14 years old	Low risk	No risk factors for VTE	Early ambulation
		At risk	≥1 risk factor (excluding immobility) or immobility without other risk factors for VTE	Mechanical prophylaxis
		High risk	Immobility with ≥1 additional risk factor for VTE	Mechanical and (strong consideration) pharmacologic prophylaxis

Hanson et al. (12)	Children admitted to the PICU after trauma	Low risk of VTE	<13 years old and ≤3 risk factors for VTE	None
		High risk of VTE with high risk of bleeding	≥13 years old or <13 years old with ≥4 risk factors for VTE and ≥1 risk factor for bleeding	Mechanical prophylaxis and surveillance ultrasound
		High risk of VTE with low risk of bleeding	≥13 or <13 years old with ≥4 risk factors for VTE and no risk factor for bleeding	Mechanical and pharmacologic prophylaxis

Meier et al. (14)	Children 10–17 years old	Low risk	No risk factors for VTE	Early ambulation and mitigation of risk factors
		Moderate risk	≥1 risk factor for VTE (excluding immobility) or immobility with ≤1 risk factor for VTE	Mechanical prophylaxis
		High risk	Immobility with ≥2 risk factors	Mechanical and pharmacologic (to be considered) prophylaxis

Mahajerin et al. (13)	Children >12 years old	Low risk	No risk factors for VTE	Early ambulation
		Moderate risk	Combinations of risk factors for VTE determine moderate versus high risk	Mechanical prophylaxis
		High risk		Mechanical and pharmacologic prophylaxis

*VTE, venous thromboembolism*.

**Table 2:** **Summary of factors used in the stratification of risk of venous thromboembolism (VTE) in the different guidelines**.

Risk factors	Braga and Young (10)	Raffini et al. (11)	Hanson et al. (12)	Meier et al. (14)	Mahajerin et al. (13)
Central venous catheter	√	√	√	√	√
Exogenous estrogen	√	√	√	√	√
Immobility	√	√	√	√	√
Inflammatory disease		√	√	√	√
Lower extremity trauma	√	√	√	√	
Obesity	√	√		√	√
Prior VTE		√	√	√	√
Infection		√		√	√
Malignancy	√		√	√	
Spinal cord injury		√	√	√	
Thrombophilia		√	√	√	
Burns	√	√			
Cardiac disease	√				√
Family history of VTE		√			√
Lower extremity surgery		√		√	
Mechanical ventilation	√				√
Nephrotic syndrome		√		√	
Pregnancy	√	√			
Admission to the intensive care unit					√
Asparaginase therapy				√	
Cardiopulmonary resuscitation			√		
Hyperosmolar therapy				√	
Inotropes			√		
Low Glasgow Coma Score			√		
Major surgery	√				
Parenteral nutrition					√
Prolonged hospital stay					√
Sickle cell disease					√
Steroids	√				

## Results

We obtained a total of 55 articles from our literature search. Of these, four articles fulfilled our criteria (10–13). An additional article was added from our personal list (14).

### Braga and Young (10)

Braga and Young proposed a pediatric thromboprophylaxis flow chart based on a survey they conducted among PICUs in England and Wales, review of their cases of DVT and a formal literature review. They recommended that all children in their PICUs be assessed daily for VTE while immobile. For this, they created a risk assessment table patterned after one that is used in adults. Risk factors included CVC, pregnancy, congenital heart disease, obesity, malignancy, major trauma, massive burns, oral contraceptive pill, prolonged surgery, long term steroids, and mechanical ventilation. In low-risk children without any risk factors, early mobilization and adequate hydration were recommended. In addition to these, mechanical prophylaxis was recommended for at-risk children defined as those with any risk factor. Short-term CVCs should be removed within 24 h unless a physician documents that it should remain. Anticoagulation with LMWH should be considered for children with burns. However, a consultant had to make the decision to anticoagulate. Compliance with the protocol and frequency of DVT or bleeding were not reported.

### Raffini et al. (11)

As part of a patient safety and quality improvement initiative that began in response to several adolescent and young adults with hospital-acquired lower extremity DVT and PE, Raffini et al. used local data, published adult guidelines, and expert consensus to develop institutional recommendations for VTE prophylaxis. This initiative was targeted at hospitalized children ≥14 years old. In their guideline, the mobility status played an important criterion for risk stratification. Children ≥14 years old who were mobile during their stay were considered low risk and ambulation was encouraged. Children ≥14 years who had altered mobility were assessed for additional prothrombotic risk factors (i.e., obesity, estrogen, major surgery, burns, trauma, thrombophilia, nephrotic syndrome, prior VTE, inflammatory disorders, and infection). They were considered at risk for VTE if none of the additional prothrombotic risk factors were present, and high risk if at least one additional risk factor was present. Mechanical prophylaxis was recommended for at-risk children, while children who were at high risk were recommended to receive mechanical and/or pharmacologic prophylaxis. Contraindications for pharmacologic prophylaxis were included in the guideline. Dosing strategies for anticoagulation were largely extrapolated from adult recommendations (30 mg enoxaparin twice daily for high-risk orthopedic surgery patients, and 40 mg enoxaparin daily for medical patients for those >60 kg). The recommendations also included placement of sequential compression devices in all children ≥14 years old undergoing surgical procedures >45 min duration.

Compliance with the recommendations improved from 22 to >80% over the 4-year study period. A follow-up prospective study evaluating bleeding in 89 patients who received enoxaparin reported two major bleeding events and five minor bleeding events, all in children who underwent major orthopedic surgery (15). Therefore, the risk of major bleeding in the orthopedic surgery patients was 4% (2/51) and 0% (0/38) in the remaining patients. No child developed a non-CADVT.

### Hanson et al. (12)

Hanson et al. reported on a guideline for VTE prophylaxis in children admitted to the PICU after trauma. Based on the guideline, children were classified into three categories: high risk of VTE and without high risk of bleeding, high risk of VTE and with high risk of bleeding, and low risk of VTE. Risk factors for VTE were projected prolonged immobility, low Glasgow Coma Scale score, presence of CVC, spinal cord injury, complex lower extremity fracture, operative pelvic fracture, use of inotropes, cardiopulmonary resuscitation, exogenous estrogen, chronic inflammatory state, history of previous DVT, known thrombophilia, and current malignancy. High risk of VTE was defined as age >13 years or age <13 years with four or more risk factors for VTE. Low risk of VTE was age <13 years and three or fewer risk factors for VTE. Risk factors for bleeding were intracranial bleed, solid organ injury, planned surgical intervention, or invasive procedure in the next 24 h, heparin allergy, high risk of severe bleeding, and renal failure. The presence of one or more risk factors for bleeding classified the patient as high risk of bleeding. For patients at high risk of VTE and without high risk of bleeding, pharmacologic prophylaxis with LMWH and mechanical prophylaxis were recommended. For patients at high risk of VTE and with high risk of bleeding, only mechanical prophylaxis was recommended. However, screening ultrasound for VTE had to be performed if the child was still in the PICU on hospital day 7. Therapeutic heparin was started if the ultrasound showed VTE and if the bleeding risk has diminished. Children at low risk of VTE were not recommended to receive prophylaxis nor surveillance ultrasound be performed.

The authors reported a compliance with the guideline of 93%. Of 169 children, 60 were high risk of VTE and high risk of bleeding, 16 high risk of VTE and without high risk of bleeding, and 93 low risk of VTE. A total of three (2%) children developed asymptomatic DVT detected on surveillance ultrasound. This was lower than the 5% with VTE prior to implementation of the guidelines. There were no bleeding complications.

### Meier et al. (14)

Meier et al. proposed a guideline for use in hospitalized children 10–17 years old. The recommendations were developed after a thorough review of the literature. Based on the guideline, a child is considered at low risk of VTE if the child was not expected to have prolonged altered mobility and had no risk factors. A child was at moderate risk of VTE if the child was not expected to have prolonged altered mobility but had risk factor for VTE, or was expected to have prolonged altered mobility but at most one risk factor for VTE. High risk of VTE was when prolonged altered mobility was expected and with at least two risk factors for VTE. Risk factors for VTE include bloodstream infection, CVC, history of VTE, hyperosmolar state, inflammatory diseases, asparaginase, estrogen use, obesity, oncologic diagnosis, hip or knee reconstruction, nephrotic syndrome, thrombophilia, multiple lower extremity long bone fracture, complex pelvic fractures, and spinal cord injury. For the low-risk group, early ambulation was encouraged and risk factors mitigated. For the moderate-risk group, use of mechanical prophylaxis was recommended in addition to the recommendations for the low-risk group. For the high-risk group, pharmacologic prophylaxis should also be considered. Absolute and relative contraindications to pharmacologic prophylaxis were listed in the guideline. The authors did not report the compliance with the guideline nor the frequency of DVT and bleeding with the use of the guideline.

### Mahajerin et al. (13)

Mahajerin et al. developed a guideline for use in hospitalized children >12 years old. For children younger than 12 years old, VTE prophylaxis was considered on an individualized basis. Based on literature review in children and adults, and statistical modeling, they ranked the association of different factors with VTE and grouped them into two tiers. Tier 1 risk factors were those that retained statistical significance in a multivariable regression model. This included prolonged immobilization, estrogen therapy, and prolonged hospital stay. Additional factors added in tier 1 were autoimmune disease with antiphospholipid antibody positivity and acute flare, dilated cardiomyopathy, atrial fibrillation, single-ventricle physiology, palliative surgical shunts, cystic fibrosis with *B. cepacia* infection, diabetic ketoacidosis, inflammatory bowel disease with acute flare, sickle cell anemia, history of VTE, known thrombophilia, and myocardial infarction in a first- or second-degree relative <50 years old. Tier 2 factors were statistically significant in the bivariate analysis but not in the multivariable regression model. This included bacteremia, obesity, chronic parenteral nutrition, initial PICU admission, mechanical ventilation, and other serious bacterial infections. The combination of these risk factors identified a child’s risk group. Only ambulatory children without risk factors were included in the low-risk group. Non-ambulatory children with or without a CVC but not any other risk factor classified a child as moderate risk. Presence of any other risk factors elevated the category to high risk. In the low-risk group, only ambulation was recommended. In the moderate-risk group, mechanical prophylaxis was also recommended. Mechanical and pharmacologic prophylaxis were recommended in the high-risk group unless with contraindications. Separate lists of contraindications for mechanical and pharmacologic prophylaxis were included in the guideline.

For the first 17 months that the guideline was implemented, an average of 69% of 149 qualified patients were screened. Compliance with the guidelines was not reported. However, none of the screened patients developed VTE compared to three cases in the 12 months prior to the guidelines. No bleeding events were reported with the use of enoxaparin.

## Discussion

Although the rate is increasing, VTE is still far less common in children than in adults (1, 16). Thus, current recommendations for treatment of VTE in children are largely extrapolated from adult studies (17). Similarly, many of the prevention strategies discussed above were based upon adult guidelines and expert opinion, in response to adolescents who developed hospital-acquired VTE as well as a national initiative implemented in 2008 by the Joint Commission to focus attention on VTE prevention (18). In this systematic review, we identified five guidelines for the prevention of VTE in children. The guidelines were mainly targeted at adolescents and used various risk-stratification approaches. Over 20% of patients hospitalized in children’s hospitals are 14 years or older and 6% are 18 years or older (11). Furthermore, hospitalized adolescents may have multiple risk factors for VTE, rivaling those of their adult counterparts. However, adolescents or young adults with hospital-acquired, non-CADVT account for a relatively small proportion of VTE in children, while the majority of thrombotic events occur in younger children and are associated with CVC (11). Therefore, it is important to recognize that adult thromboprophylaxis guidelines are based upon the proven efficacy of anticoagulants to reduce the rate of lower extremity DVT, and not necessarily CADVT.

In a recent survey of pediatric hemostasis and thrombosis experts in North America, Badawy et al. showed that approximately one-third and one-half of their respondents reported having guidelines for pharmacologic and mechanical prophylaxis against DVT, respectively, in their hospitals (19). The majority of the respondents did not support the adoption of universal pharmacologic prophylaxis. There was significant variability on which risk factors influenced the decision to provide pharmacologic prophylaxis. Our findings are consistent with this survey. None of the published guidelines recommended universal pharmacologic prophylaxis. While age and immobility were consistent determinants of the risk of VTE across guidelines, there was significant variability in the other risk factors considered. It is interesting to note that despite being the most common cause of DVT in children, the presence of CVC was not a prominent factor in the risk-stratification approaches. This is likely related to the negative RCTs on pharmacologic prophylaxis against CADVT in children (9). In low-risk children, ambulation was the only recommended intervention. Mechanical prophylaxis was recommended for moderate risk while pharmacologic and mechanical prophylaxis were recommended for the high-risk group if there were no contraindications to pharmacologic prophylaxis. The choice of prevention strategy likely reflected the perceived safety of mechanical prophylaxis, despite its reduced efficacy compared to pharmacologic prophylaxis, in children, based on data from adults (5–7).

Randomized controlled trials are an ideal way to determine the efficacy of an intervention. However, the conduct of RCTs of thromboprophylaxis in children has been difficult. A major problem is the low numbers of clinically apparent VTE in children (9). In hospitalized children, the rate is only 34–58 cases per 10,000 admissions (1). Thousands of children and multiple centers will be needed to successfully complete an RCT that will detect a clinically significant reduction in VTE. To increase the frequency of the outcome, and thus decrease the estimated sample size, ultrasound diagnosed DVT have been recommended and used as outcomes in RCTs in adults and children (5–7, 9, 20). Because some subjects have asymptomatic DVT, using routine ultrasound in a study increases the event rate compared to using symptoms to guide which patients are evaluated for DVT. The increased event rate leads to the need for a smaller sample size to achieve the same power to detect a treatment difference. Aside from the convenience in lowering the sample size, the use of ultrasound diagnosed DVT as outcome minimizes the risk of ascertainment bias in detecting DVT by physical examination alone (20). Venography was frequently used to diagnose DVT in adult studies (21). Because of difficulties in performing venography in children, its use has been supplanted by ultrasonography. Some practitioners question the use of ultrasound-diagnosed DVT as outcome. In a survey of pediatric critical care physicians, most stated that asymptomatic DVT diagnosed by radiologic imaging alone are not clinically significant (22). Long-term outcomes are needed to determine whether asymptomatic DVT is a clinically relevant outcome in children (23).

If RCTs will be performed to determine the efficacy of prophylaxis against DVT in children, innovative study designs are needed. For example, a risk-stratified approach that mirrors the published guidelines may need to be incorporated into the study design. Bayesian study design with adaptive randomization is a potential alternative to determine the differential effect of mechanical and/or pharmacologic prophylaxis (24). This design has the advantage of dropping treatment arms that are likely to be futile then direct subjects to promising treatment. Sample size decreases because more subjects are enrolled in fewer treatment arms. Quasi-experimental designs, such as stepped wedge trials, should also be considered. Such a design can enhance participation because each center is guaranteed to get the intervention at some point (25).

In lieu of RCTs, other sources of evidence are needed to inform our practice on the prevention of VTE in children. Guidelines can provide some of this information because their consistent use can minimize variability in practice and increase the replicability of results (26). However, it is crucial that data are collected accurately. Of the five guidelines reviewed, only two provided data on compliance and three provided data on the frequency of DVT and bleeding (11–13). Each center on its own is unlikely to obtain sufficient numbers of children to prove any statistically significant difference. Meta-analysis of data from each of the centers might be a useful approach to strengthen conclusions about the efficacy of VTE prevention strategies. It may be possible to perform such analysis because of similarities in the guidelines, particularly those of Raffini et al., Meier et al., and Mahajerin et al. (11, 13, 14). Comparable historical controls would be required to make the analysis robust. This would require careful thought to identify appropriate controls. Finally, the Solutions for Patient Safety network is collecting high-quality data on patients with VTE and strategies are being developed to reduce VTE (8). This quality improvement initiative has the potential to identify effective VTE prevention strategies.

In conclusion, evidence is lacking on the right approach to the prevention of VTE in children. RCTs, which are the gold standard, are very difficult to conduct. Innovative designs are needed to successfully complete these RCTs. Guidelines have been developed despite the paucity of evidence to help with patient management. These guidelines can be a source of valuable information to inform our practice.

## Author Contributions

EVF and LR conceived and designed, collected, analyzed, and interpreted the guidelines, and co-wrote the first draft of the manuscript. Both authors revised the manuscript for important intellectual content, approved this version of the manuscript, and took full responsibility for the manuscript.

## Conflict of Interest Statement

The authors declare that the research was conducted in the absence of any commercial or financial relationships that could be construed as a potential conflict of interest.
